# Polychlorinated biphenyls and breast cancer: evidence, mechanisms, and risk management

**DOI:** 10.3389/fcell.2026.1744112

**Published:** 2026-02-25

**Authors:** Shuaiyang Zhang, Hongliang Cao, Runzhou Lian, Ying Liu, Yuting Zhu, Xinxin Gui, Tianzi Kong, Aiping Shi

**Affiliations:** 1 Department of Breast Surgery, General Surgery Center, The First Hospital of Jilin University, Changchun, China; 2 Department of Urology, The First Hospital of Jilin University, Changchun, China

**Keywords:** breast cancer, epigenetic locking, metabolic reprogramming, polychlorinated biphenyls, precision prevention

## Abstract

Epidemiological inconsistencies currently obscure the causal link between polychlorinated biphenyls (PCBs) and breast cancer. This review synthesizes multi-disciplinary evidence to characterize PCBs not as passive exposure correlates but as active drivers of tumorigenesis via a metabolic–oxidative–epigenetic axis. We examine how lipophilic congeners accumulate in adipose reservoirs, extending toxicity across the life course beyond distinct susceptibility windows. Mechanistically, receptor crosstalk between the aryl hydrocarbon receptor (AhR) and the estrogen receptor (ER) triggers mitochondrial dysfunction and inhibits ten–eleven translocation (TET) enzyme activity, creating an oxidative state that establishes epigenetic locking of tumor suppressor genes. We propose subtype-specific evolutionary trajectories: postulating that dioxin-like congeners drive AhR-mediated stemness in triple-negative phenotypes, whereas non-dioxin-like mixtures impose an oxidative bottleneck that facilitates the acquisition of therapeutic resistance. Finally, we propose an integrated risk management framework connecting upstream environmental remediation (e.g., bio-nano systems) with downstream clinical stratification and gut–liver axis interventions. This framework establishes a biological foundation for understanding PCB-induced malignancy while defining actionable pathways for exposure-informed precision prevention.

## 1 Introduction

Breast cancer constitutes the predominant malignancy and a primary cause of cancer-related mortality among women globally. According to GLOBOCAN 2022, the burden of disease exhibits a stark epidemiological dichotomy: although incidence peaks in regions with a very high human development index (HDI), mortality is disproportionately concentrated in lower-HDI settings (Filho et al., 2025; Kim et al., 2025; Li et al., 2024). Although reproductive history, hormonal status, and lifestyle factors are established determinants, they do not fully account for these disparities or the increasing global incidence (Coles et al., 2024). This explanatory gap strongly implicates environmental pollutants, particularly polychlorinated biphenyls (PCBs), as underappreciated drivers within the etiological landscape (Leng et al., 2016).

The substantial molecular heterogeneity of the disease compounds the complexity of addressing this burden. Breast cancer is not a singular entity but a spectrum of intrinsic subtypes—luminal A, luminal B, HER2-enriched, and basal-like/triple-negative (TNBC)—each defined by distinct gene-expression profiles and therapeutic sensitivities (Malhotra et al., 2014; Perou et al., 2000). Although endocrine and anti-HER2 therapies have improved outcomes for hormone-receptor-positive disease, the clinical recalcitrance of TNBC and advanced metastatic stages remains a critical challenge (Xiong et al., 2025; Smolarz et al., 2022). This therapeutic ceiling implies that extrinsic exposures may intersect with intrinsic tumor biology to drive disease progression (Parada et al., 2020; Qiu et al., 2020). Emerging evidence implicates persistent pollutants—particularly PCBs—as active modulators exacerbating tumor aggression beyond simple genetic initiation (Qin et al., 2021; Wang et al., 2018). Consequently, this review moves beyond hazard identification to prioritize actionable risk mitigation. We propose a multi-scalar intervention framework that bridges environmental remediation with clinical stratification, thereby advancing the paradigm of exposure-informed precision prevention.

### 1.1 Literature search strategy

A systematic search of PubMed/MEDLINE, Web of Science, and Scopus identified the literature published through January 2026. The core syntax combined (“polychlorinated biphenyls” OR PCBs) with (“breast cancer” OR “breast neoplasms”). This strategy extended to mechanistic, exposure, and interventional domains. Specific queries targeted “aryl hydrocarbon receptor” (AhR), “epigenetic locking,” and “signal transduction” for pathways; “mixture analysis,” “quantile g-computation,” and “life-course exposure” for exposure dynamics; and “gut–liver axis,” “remediation,” and “risk stratification” for integrated management. Selection was limited to English-language publications, prioritizing original research and meta-analyses that define congener-specific effects. Manual screening of reference lists supplemented the electronic search to ensure coverage.

## 2 PCBs and their metabolites

PCBs comprise a family of 209 theoretical congeners defined by the degree and position of chlorine substitution on the biphenyl ring (NIH, 2016). Toxicologically, they are dichotomized into dioxin-like (DL-PCBs) and non-dioxin-like (NDL-PCBs) groups, based on their ability to adopt a coplanar conformation and bind the AhR (NIH, 2016; Safe, 1994). However, relying solely on this structural dichotomy often obscures the nuanced biological complexity observed in population studies. Therefore, to bridge the gap between mechanistic toxicity and epidemiological risk, we integrate the functional grouping proposed by Wolff et al. (1997). As detailed in Table 1, this framework stratifies congeners into three distinct clusters that align with their dominant toxicological modes of action: potentially estrogenic (Group I, comprising primarily low-chlorinated NDL-PCBs), anti-estrogenic/dioxin-like (Group II, corresponding to DL-PCBs), and enzyme-inducing (Group III, encompassing primarily high-chlorinated NDL-PCBs) (NIH, 2016; Wolff et al., 1997). This synthesis allows for a more precise characterization of how structural distinctions dictate divergent—and often opposing—evolutionary trajectories in mammary carcinogenesis.

**TABLE 1:** Classification, toxicological profiles, and risk assessment of PCB congeners in breast cancer.

Functional group and representative congeners	Physicochemical and toxicological profile	Epidemiological association (OR, 95% CI)	Risk assessment and evidence strength
Group I: potentially estrogenic (low-chlorinated NDL-PCBs); e.g., PCBs 28 and 52	Properties: high volatility, lower persistence, and shorter half-life (Beyer and Biziuk, 2009) Mechanism (Wolff et al., 1997) (1) Weak ERα agonism (estradiol-mimetic) (2) Weak phenobarbital inducers	Pooled group-level	Risk level: Low/inconclusive Evidence: Weak and inconsistent Note: High volatility leads to retrospective exposure misclassification (Zhang et al., 2015)
		OR ≈ 1.10 (95% CI: 0.97–1.24) (Zhang et al., 2015) Congener-specific associations are generally null (Zhang et al., 2015)	
Group II: dioxin-like/anti-estrogenic (high-chlorinated, DL-PCBs); e.g., PCBs 77, 118, 126, and 156	Properties: planar structure (coplanar) and high TEF values (NIH, 2016) Mechanism (1) Potent AhR agonism (Chatterjee and Banerjee, 2023) (2) Induction of CYP1A1/1B1 (Chatterjee and Banerjee, 2023) (3) Anti-estrogenic crosstalk (Leng et al., 2016)	Pooled group-level OR 1.23 (95% CI: 1.08–1.40) (Zhang et al., 2015) PCB 118 individual results are often mixed (Leng et al., 2016)	Risk level: Moderate/plausible Evidence: Mechanistically strong but epidemiologically varied Context: Anti-estrogenic crosstalk between AhR and ER pathways suggests risk heterogeneity (e.g., potential specificity for ER-negative subtypes), although epidemiological evidence remains limited (Zhang et al., 2015)
Group III: phenobarbital-type inducers (high-chlorinated, NDL-PCBs); e.g., PCBs 99, 138, 153, 180, 183, and 187	Properties: extreme lipophilicity and highest bioaccumulation in adipose (Beyer and Biziuk, 2009) Mechanism (Grimm et al., 2015) (1) Oxidative stress (ROS) (2) CYP2B enzyme induction and reactive metabolites	Congener-specific meta-analysis (Leng et al., 2016) (1) PCB 99 ▲ **OR: 1.36 (1.02–1.80)** (2) PCB 183 ▲ **OR: 1.56 (1.25–1.95)** (3) PCB 187 ▲ **OR: 1.18 (1.01–1.39)** (4) PCB 138 △ 1.08 (0.99–1.17) (5) PCB 153 ○ 1.04 (0.81–1.34) (6) PCB 180 ○ 1.02 (0.81–1.29)	Risk level: Significant (specific congeners) Evidence: Statistically significant in meta-analysis for PCB 99/183/187 (5) Structure–toxicity relationship: The null results for the highly abundant PCBs 153/180 suggest that risk is not dose-dependent but linked explicitly to CYP2B enzyme induction (observed in PCB 99/183) (Leng et al., 2016)
Metabolites: hydroxylated PCBs (OH-PCBs) e.g., 4-OH-CB107 and 3-OH-CB153	Properties: Increased polarity and retained in blood (protein-bound) (Quinete et al., 2014) Mechanism (Grimm et al., 2015) (1) Endocrine activity (Modulation of ER-mediated transcription)	Stable pooled estimates unavailable due to panel heterogeneity (Quinete et al., 2014) Detected in serum/adipose of breast cancer patients (Nomiyama et al., 2010)	Risk level: Emerging concern Status: Biologically potent but clinically under-monitored Gap: Represents the “active” form of toxicity often missed in parent-compound studies (Grimm et al., 2015)

Symbols: ▲ significant association (*p* < 0.05); △ borderline trend; ○ null association (*p* ≥ 0.05).

Abbreviations: AhR, aryl hydrocarbon receptor; CI, confidence interval; CYP, cytochrome P450; DL-, dioxin-like; ERK, extracellular signal-regulated kinase; ER, estrogen receptor; MAPK, mitogen-activated protein kinase; NDL-, non-dioxin-like; OR, odds ratio; PCB, polychlorinated biphenyl; ROS, reactive oxygen species; TEFs, toxic equivalency factors.

Data sources: Group-level pooled ORs are derived from the study by Zhang et al. (2015), and congener-specific ORs are derived from the meta-analysis by Leng et al. (2016). Classification is based on the Wolff/Toniolo functional grouping. The structural/TEF context for DL-PCBs aligns with the International Agency for Research on Cancer (IARC) Monograph 107 (NIH, 2016).

Despite these biological distinctions, all congeners share robust physicochemical characteristics. Owing to their chemical stability, high lipophilicity, and flame-retardant and electrical-insulating properties, PCBs were historically manufactured and widely used in industrial applications, including as insulating fluids in transformers, dielectric fluids in capacitors, and plasticizers (Safe, 1994). Despite global restrictions on commercial production enacted decades ago, PCBs persist as ubiquitous environmental contaminants (Zhu et al., 2022). Their consistent detection across air, water, soils, and sediments reflects ongoing releases from historical repositories, improper disposal, and leakage from aging infrastructure (Beyer and Biziuk, 2009). Fundamentally, their recalcitrance, capacity for long-range atmospheric transport, and bioaccumulative potential in food webs drive their global distribution, posing sustained risks to ecological integrity and human health (Zhu et al., 2022; Beyer and Biziuk, 2009).

Humans are exposed to PCBs through dietary intake (Pruvost-Couvreur et al., 2021), inhalation (Deen et al., 2025), dermal contact (Ertl and Butte, 2012), and transplacental and lactational transfer (Zhang et al., 2021; Balalian et al., 2025). These compounds are absorbed by aquatic organisms (e.g., fish and crustaceans) via particle adsorption in water bodies, accumulate in adipose tissue, and enter the food chain. Human consumption of contaminated animal-based foods leads to the long-term accumulation of PCBs in body fat due to their high lipophilicity and metabolic resistance (Beyer and Biziuk, 2009). These compounds can be transferred to the next generation via the placenta and breast milk (Wang et al., 2021). Epidemiological studies have linked PCBs to various systemic diseases (Symeonides et al., 2024), including cardiovascular diseases (Liu et al., 2025), endometriosis (Cano-Sancho et al., 2019), breast cancer (Sánchez-Ocaña and Ruiz de Porras, 2025), type 2 diabetes (Li et al., 2023), prostate cancer (Zheng et al., 2024), and chronic kidney disease (Chai et al., 2025).

The link between PCB exposure and breast cancer has garnered increasing scrutiny. However, carcinogenic potential is not uniform. Table 1 synthesizes this heterogeneity, aligning functional toxicity with epidemiological risk levels. Meta-analyses have reported robust positive associations for specific congeners such as PCBs 99 and 183, while evidence for PCB 187 suggests a potential risk. In contrast, findings for others (e.g., PCBs 138, 153, and 180) remain weak or inconsistent across systematic reviews (Leng et al., 2016; Liu H. et al., 2023). Distinct from these epidemiological patterns, DL-PCBs act via more complex and potent AhR-mediated mechanisms (Chatterjee and Banerjee, 2023), while hydroxylated metabolites (OH-PCBs) act as potent endocrine disruptors (Wan et al., 2022; Grimm et al., 2015). In contrast, epidemiological findings for Group I are sparse and mostly null, with most studies failing to demonstrate consistent associations with the breast cancer risk (Zhang et al., 2015; Negri et al., 2003). However, the absence of epidemiological consensus does not preclude biological toxicity, particularly regarding biotransformation products. PCB metabolism, primarily mediated by hepatic cytochrome P450 enzymes, generates a diverse array of reactive and stable metabolites, including OH-PCBs, quinones, sulfate esters, and methylsulfonyl derivatives (MeSO_2_-PCBs) (Grimm et al., 2015). Evidence indicates that these metabolites contribute significantly to the observed carcinogenic, endocrine-disrupting, and neurotoxic risks associated with PCB exposure (Grimm et al., 2015). Mechanistically, PCBs are implicated in breast cancer pathogenesis through AhR-mediated toxicity, epigenetic modifications, oxidative stress, and interference with estrogen receptor (ER) signaling pathways (Chatterjee and Banerjee, 2023; Wan et al., 2022; Singh, 2024). The heterogeneity of these mechanisms stems from differences among PCB congeners (as summarized in Table 1).

## 3 Multilevel evidence linking PCB exposure to the breast cancer risk

The distinct toxicological profiles of PCB congeners dictate their biological risk, yet establishing causality in human populations remains methodologically challenging. Inconsistencies in epidemiological data often stem from non-linear dose responses and uncaptured exposures during critical windows of susceptibility. Resolving these disparities requires synthesizing evidence across disciplines. In this study, we integrate methodological refinements with experimental mechanisms and clinical outcomes to characterize specific congeners as active drivers of breast carcinogenesis.

### 3.1 Non-linearity, windows of susceptibility, and exposure assessment challenges

A “high-dose paradox” complicates epidemiological interpretation, creating a divergence between occupational and environmental findings. High-exposure cohorts, including capacitor workers, frequently show null or inverse associations (SIR = 0.81; 95% CI: 0.72–0.92) (Silver et al., 2009). This discrepancy challenges the assumption of linearity. Vandenberg et al. (2012) noted that endocrine-disrupting chemicals, including PCBs, often exhibit non-monotonic dose–response curves with U- or inverted-U-shaped profiles. Mechanistically, low environmental doses may saturate high-affinity receptors (e.g., ER and AhR) to drive mitogenic signaling, whereas high industrial doses can induce cytotoxicity or receptor downregulation, thereby masking carcinogenic effects (Vandenberg et al., 2012). Consequently, null findings in high-exposure settings do not preclude risks at lower, biologically relevant concentrations (Vandenberg et al., 2012).

**FIGURE 1:**
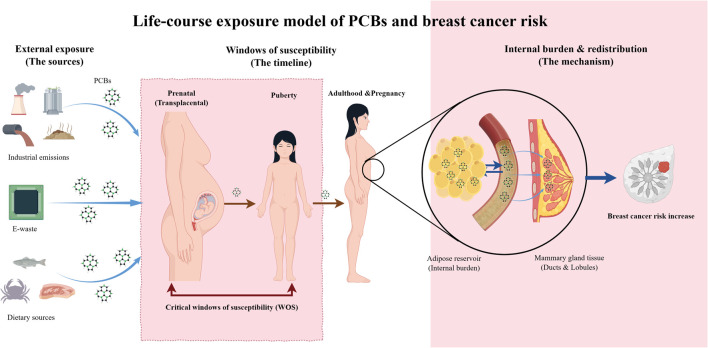
Integrated life-course exposure dynamics of PCBs in breast carcinogenesis.

Temporal ambiguity compromises exposure assessment, particularly in case–control designs where post-diagnostic sampling risks “reverse causation” and fails to capture antecedent exposures (Rodgers et al., 2018). The “Windows of Susceptibility” framework locates mammary gland vulnerability within specific periods of rapid structural remodeling: prenatal development, puberty, and pregnancy. Consequently, the proposed life-course model (Figure 1) shifts focus from static measurement to cumulative process. Under the Developmental Origins of Health and Disease (DOHaD) hypothesis, exposure during these critical windows establishes a latent carcinogenic potential. Although this trajectory is empirically validated for *in utero* DDT exposure (Cohn et al., 2015), a similar pathway is postulated for PCBs, characterized by bioaccumulation in adipose tissue and subsequent mobilization to the mammary gland (Zeinomar et al., 2020). Standard adult measurements frequently overlook these etiological events. Cohn et al. (2012) circumvented this limitation by analyzing archived postpartum serum, assessing PCB burdens during a biologically relevant pre-diagnostic window to minimize metabolic interference.

Beyond temporal dynamics, accurate risk estimation requires robust statistical methods to account for both physiological variability and the complexity of chemical mixtures. Lipid adjustment methods significantly affect the validity of risk estimation. Schisterman et al. (2005) demonstrated that dividing PCB concentrations by serum lipids introduces statistical bias; treating lipids as independent covariates is a more robust method to disentangle biological variability from measurement artifacts. Furthermore, relying on single-congener models limits the interpretation of real-world toxicity. Human exposure involves complex combinations of lipophilic pollutants that traditional regression cannot adequately address due to multicollinearity. Advanced frameworks, such as the quantile g-computation used by Parada et al. (2021), have revealed mixture-specific risks and racial disparities that are often obscured by single-pollutant models. Hao et al. (2025) emphasized that future assessments must adopt such high-dimensional strategies to capture synergistic effects. Finally, residual confounding from reproductive factors remains an additional concern. As noted by Gatto et al. (2007), lactation serves as a primary excretion route for maternal PCBs, necessitating careful adjustment to disentangle the protective effect of breastfeeding from lower PCB body burdens.

Figure 1 depicts the progression from external sources—dietary, atmospheric, and industrial—to a cumulative internal burden. Under the DOHaD hypothesis, exposure during critical windows of susceptibility (prenatal and pubertal development) initiates a latent carcinogenic potential. The adipose reservoir functions as a dynamic interface, driving the bioaccumulation and subsequent mobilization of lipophilic congeners to the mammary epithelium. This redistribution mechanism links historical exposure to late-onset malignancy.

### 3.2 Experimental support for the tumor-promoting properties of PCBs and their metabolites

The methodological limitations of observational epidemiology highlight the indispensable role of experimental models in establishing biological plausibility. Where human cohort data remain heterogeneous, *in vitro* and *in vivo* studies consistently reveal the tumor-promoting phenotypes driven by PCBs. Controlled laboratory settings effectively isolate these carcinogenic mechanisms from complex confounders, clarifying the direct causality often masked in population studies. Wang et al. (2018) demonstrated that the quinone metabolite PCB29-pQ increased migration and invasion of breast cancer cells (MDA-MB-231 and 4T1-luc) and augmented lung and liver metastases in murine models. Building on this, Qin et al. (2021) further revealed that PCB29-pQ activated Wnt/β-catenin signaling in MDA-MB-231 cells, induced cancer stem-like traits, upregulated CD44, Sox2, and Nanog, and promoted epithelial–mesenchymal transition (EMT). In a companion study, the same group reported that PCB29-pQ upregulates GLUT1 to engage the GLUT1/integrin-β1/Src/FAK signaling axis. This activation enhances aerobic glycolysis, thereby facilitating cell migration and invasion. Corroborating these *in vitro* findings, *in vivo* bioluminescence imaging revealed intensified pulmonary and hepatic signals, indicative of an increased metastatic burden (Qin et al., 2022).

Regarding mixture effects, Liu et al. (2025) reported that a low-dose equipotent mixture of seven NDL-PCBs upregulated reactive oxygen species (ROS) and the Rho-associated kinase (ROCK)–myosin light chain axis. This activation significantly enhanced metastatic properties, specifically cell motility, in both ER-positive MCF-7 and ER-negative MDA-MB-231 cell lines. These findings suggest that NDL-PCBs drive tumor progression via an ER/AhR-independent mechanism (Aziz et al., 2010). In a pivotal *in vivo* study, Nesaretnam et al. (1998) corroborated these metastatic propensities using a DMBA-induced rat model. Exposure to 3,3′,4,4′-tetrachlorobiphenyl (TCB) accelerated tumor onset independent of dietary fat content. Although a high-fat diet increased tumor multiplicity, TCB exposure was the decisive factor driving pathological aggression. Notably, the acquisition of an invasive histological phenotype was almost exclusively restricted to TCB-treated animals (12 of 13 invasive carcinomas), distinguishing congener-driven malignancy from dietary promotion (Nesaretnam et al., 1998).

Collectively, these experimental findings provide robust mechanistic support for the observed epidemiologic associations and highlight the need to consider interactions between PCB exposure and other risk factors, such as diet. These consistently observed phenotypes, particularly metabolic reprogramming and acquired stemness, provide cellular evidence for the integrated molecular network, as detailed in the subsequent section.

### 3.3 PCBs load in blood and adipose tissue and breast cancer staging/risk

Despite these assessment challenges, epidemiological data—particularly from high-exposure and longitudinal cohorts—consistently identify PCBs as significant contributors to breast cancer incidence. For instance, in the highly exposed Greenland Inuit population, Wielsøe et al. (2017) reported that high concentrations of total PCB (ΣPCB) and specific congeners (PCB 138, PCB 153, and PCB 170) were significantly associated with an increased breast cancer risk (ORs for the highest tertile: 2.43–2.69). Critically, recent longitudinal data have expanded this risk landscape beyond dietary intake to atmospheric exposure. In the French E3N cohort, Deygas et al. (2021) identified a dose-dependent relationship between long-term atmospheric exposure to PCB153 and breast cancer risk, highlighting inhalation as an underappreciated driver of environmental carcinogenesis.

Beyond incidence, internal PCB burden is strongly implicated in altering molecular phenotypes and clinical progression. Adipose tissue—the primary reservoir for these lipophilic compounds—functions as a dynamic interface, redistributing stored congeners to the mammary gland and contributing to local pathogenicity (Qiu et al., 2020; He et al., 2017). In a study of 223 women, He et al. (2017) reported that ΣPCB levels in breast adipose tissue were positively correlated with clinical stage (*p* = 0.036), with the highest concentrations observed in patients with stage III–IV disease. Additionally, a non-monotonic dose–response relationship was observed between PCB burden and ER expression, suggesting interference with estrogen signaling (He et al., 2017).

Tumor aggressiveness depends on the exposure profile rather than solely on the cumulative burden. Specific congeners drive distinct molecular phenotypes. Qiu et al. (2020) reported that neurotoxic PCB28 levels were significantly correlated with HER2 positivity and larger tumor size, while the CYP-inducer PCB153 was associated with elevated Ki-67 expression. Notably, the total PCB burden was linked to increased VEGF-C expression and metastasis (Qiu et al., 2020). Although VEGF-C is canonically a driver of lymphangiogenesis, statistical evidence in this cohort particularly highlighted a significant association with distant metastasis rather than lymph node involvement, suggesting a complex role of cumulative PCB exposure in advanced disease progression (Qiu et al., 2020). These associations with aggressive clinicopathological features are not merely cross-sectional observations; they are predictive of clinical outcomes (Parada et al., 2020). Recent survival analyses from the Carolina Breast Cancer Study confirm that elevated circulating PCB burdens significantly correlate with increased breast cancer-specific mortality, particularly within the first 5 years post-diagnosis (Parada et al., 2020). In conclusion, elevated PCB body burden is associated not only with higher breast cancer incidence but also with more aggressive disease features, particularly advanced stage (He et al., 2017), HER2 enrichment (Qiu et al., 2020), and poorer survival outcomes in the years immediately following diagnosis (Parada et al., 2020).

### 3.4 Critical synthesis: integrating experimental mechanisms with epidemiological findings

Although experimental models consistently demonstrate carcinogenicity, epidemiological associations have historically remained fractured due to the methodological divergences discussed earlier. This section integrates these disparate lines of evidence to bridge the gap. As summarized in Table 2, emerging research resolves these historical inconsistencies by shifting the analytical focus from single agents to mixture effects and population heterogeneity.

**TABLE 2:** Comparative analysis of study models: methodological divergences and mechanistic concordance.

Domain	Experimental model	Epidemiological study	Bridging evidence
Exposure complexity	Single agents or defined mixtures. Establishes clear causality	Chronic mixed exposure. Statistical challenges (multicollinearity)	Parada et al. (2021) utilized quantile g-computation to address collinearity, revealing significant mixture-associated risks particularly among Black women, thereby resolving the statistical masking often present in single-pollutant models
Dose sensitivity	High-dose, sub-chronic exposure. Lower sensitivity (high toxicological thresholds)	High sensitivity (adverse effects at low doses)	Muir et al. (2023) quantified the “sensitivity gap,” demonstrating that human adverse effects occur at concentrations that are orders of magnitude below animal thresholds
Toxicokinetics	High bioactivation potential. Rapid conversion of lower-chlorinated polychlorinated biphenyls into reactive metabolites (e.g., quinones) dominates the toxicological profile	Rapid clearance of non-persistent congeners masks exposure. Risk assessment is biased toward accumulated parent compounds	Grimm et al. (2015) emphasized that rapid metabolism generates reactive intermediates (e.g., quinones) with distinct genotoxic and cytotoxic potentials, thereby introducing toxicity pathways (e.g., initiation) that complement the tumor-promoting effects of persistent parent compounds
Mechanisms	AhR activation and oxidative stress. Mechanism-based causality	Genomic instability and receptor status. Outcome-based association	Yang et al. (2025) validated the clinical relevance of experimental pathways by confirming that key *in vitro* validated targets (e.g., AKT1) are significantly dysregulated in human breast tumor transcriptomes (TCGA)

Abbreviations: AhR, aryl hydrocarbon receptor; TCGA, The Cancer Genome Atlas; AKT1, AKT serine/threonine kinase 1.

To address the statistical masking observed in human studies, recent analyses have shifted focus to mixture effects. Traditional single-pollutant models often fail to account for multicollinearity (Leng et al., 2016). As highlighted in Section 3.1, by applying quantile g-computation to complex mixtures, Parada et al. (2021) revealed that PCB-associated breast cancer risk is not uniform but significantly elevated in specific racial subgroups. This suggests that previous discrepancies may stem from unmeasured heterogeneity in exposure profiles rather than a lack of biological effects.

Beyond statistical refinement, interpreting human risk requires accounting for fundamental toxicokinetic differences between species. Notably, humans exhibit adverse outcomes at body-burden orders of magnitude lower than those in rodent studies, indicating heightened sensitivity to chronic accumulation (Muir et al., 2023). Furthermore, a critical divergence exists in metabolic processing: rodent pathology is primarily driven by rapid metabolism into reactive quinones, whereas human risk assessment has traditionally relied on the bioaccumulation of parent compounds. However, as highlighted by Grimm et al. (2015), neglecting these reactive metabolites ignores a distinct toxicity pathway—specifically genotoxic initiation—that complements the tumor-promoting effects of accumulated parent compounds.

Despite these kinetic distinctions, the downstream oncogenic machinery appears remarkably conserved. Translational studies have bridged the species gap by validating experimental mechanisms directly in clinical datasets. Yang et al. (2025) confirmed that signaling nodes validated *in vitro* (e.g., AKT1) are significantly dysregulated in human breast cancer transcriptomes (TCGA dataset). This finding is pivotal: it establishes that although the route of exposure and metabolism may differ between models and humans, the ultimate molecular perturbation driving the tumor remains identical.

Consequently, this convergence of epidemiological refinement and molecular validation confirms that PCB-induced malignancy is not an artifact of experimental high-dosing but a relevant human pathology. This pathology is mediated by a specific network of interactions, as detailed in the following section.

## 4 Multiple mechanisms: an integrated network of PCB-induced carcinogenesis

**FIGURE 2:**
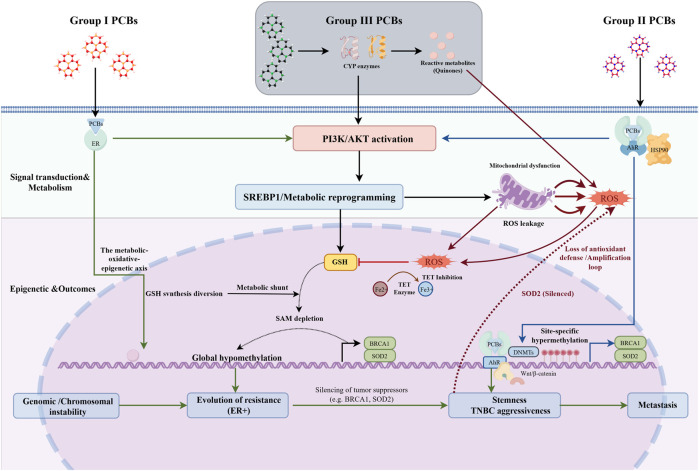
Integration of receptor crosstalk and the metabolic–epigenetic axis in PCB-induced breast carcinogenesis.

PCB toxicity initiates through strict congener-dependent molecular events. DL-PCBs predominantly activate the AhR signaling axis (Safe, 1994); in contrast, NDL-PCBs act as endocrine disruptors (Singh, 2024). Current evidence indicates a functional convergence of these pathways: AhR–ER crosstalk modifies the tumor microenvironment (Chatterjee and Banerjee, 2023), while oxidative stress drives epigenetic dysregulation (Singh, 2024). Synthesizing these findings, we propose a framework in which receptor interference triggers metabolic reprogramming, precipitating a stable, oxidative-stress-mediated “epigenetic locking.” As illustrated in Figure 2, this feedback loop links signaling aberrations to genomic instability, promoting tumorigenesis, metastasis, and therapeutic resistance.

This framework illustrates the translation of heterogeneous toxicological inputs into subtype-specific malignant evolution. The convergence of receptor signaling and metabolic reprogramming generates a chronic oxidative milieu, driving “epigenetic locking” via ten–eleven translocation (TET) inhibition and methyl donor depletion. Notably, the targeted silencing of *SOD2* closes a self-amplifying feedback loop, sustaining the oxidative pressure required to maintain this blockade. This mechanism functions as an evolutionary bottleneck, diverging tumor progression: in ER + lineages, global hypomethylation induces genomic instability and therapeutic resistance; in TNBC, constitutive AhR signaling integrates with the Wnt/β-catenin pathway to promote aggressive stemness traits.

### 4.1 Receptor crosstalk, balance disruption, and metabolic reprogramming

The biochemical and toxic responses, including carcinogenic potency, of PCBs are primarily determined by specific chlorination patterns that dictate their conformational topology and receptor affinity (Safe, 1994). Emerging evidence further suggests that these structural features may drive synergistic receptor interactions (Yang et al., 2025). In particular, estrogenic activity is predominantly associated with lower-chlorinated, ortho-substituted congeners (Group I) and their OH-PCBs (Wolff et al., 1997). Mechanistically, steric hindrance imposed by ortho-chlorines prevents these congeners from adopting the coplanar conformation strictly required for high-affinity AhR binding, thereby shifting their activity profile toward estrogen receptor signaling (Safe, 1994; Wolff et al., 1997). Instead, facilitated by this conformational restriction, these congeners adopt an estradiol-mimetic geometry and function as Erα agonists, thereby driving the transcription of proliferative genes such as *CCND1* (Sabbah et al., 1999; Korach et al., 1988). The resulting accumulation of cyclin D1 accelerates the G1/S transition, bypassing cell cycle checkpoints and fueling sustained tumor expansion (Musgrove et al., 2011).

The convergence of AhR signaling compounds this dysregulation. Coplanar dioxin-like PCBs act as high-affinity ligands for AhR (Safe and Wormke, 2003). Although historical paradigms suggested competitive antagonism between AhR and ERα, accumulating mechanistic evidence reveals a functional convergence in the tumor microenvironment (Chen et al., 2024). In breast cancer tissues, where AhR is frequently overexpressed relative to normal tissue (Mohamed et al., 2019), activated AhR engages in crosstalk with signal transducers, including ERα and c-Src kinases, to amplify mitogenic signaling beyond canonical transcriptional regulation (Chen et al., 2024). This crosstalk is synergistic, converging on shared downstream hubs such as the PI3K/AKT axis (Chen et al., 2024).

This constitutive activation of the PI3K/AKT axis serves as the molecular trigger for profound metabolic reprogramming. Prominently, sustained signaling orchestrates the upregulation of sterol regulatory element-binding protein 1 (SREBP1), the master transcriptional regulator of lipogenesis (Hajirahimkhan et al., 2025). Consequently, the cell shifts into a hyper-anabolic state, characterized by *de novo* fatty acid synthesis and enhanced aerobic glycolysis (the Warburg effect) to meet the biosynthetic demands of rapid division (Winz et al., 2023). However, this metabolic acceleration imposes a toxic cost: the overburdened mitochondrial electron transport chain inevitably leaks ROS, establishing a persistent oxidative milieu (Thakur et al., 2022). This chronic oxidative stress extends beyond mere cellular toxicity; it fosters a mutagenic environment that predisposes the genome to profound epigenetic instability.

### 4.2 Oxidative-driven epigenetic reprogramming

Metabolic activation of PCBs generates a persistent flux of ROS, transforming a metabolic byproduct into a driver of pathogenic signaling. Beyond direct genotoxicity, this oxidative milieu actively subverts the epigenetic machinery, establishing a “metabolic–oxidative–epigenetic” axis that locks the genome into a pro-tumorigenic state.

TET dioxygenases strictly require ferrous iron (Fe^2+^) as a cofactor to maintain fidelity in DNA demethylation. Elevated ROS levels disrupt labile iron homeostasis by oxidizing this cofactor to its ferric state (Fe^3+^), restricting the Fe^2+^ bioavailability essential for enzymatic turnover (Ma et al., 2025; Niu et al., 2015). Critically, this oxidative impairment of TET-mediated active erasure creates a permissive environment for aberrant DNA methylation (Niu et al., 2015). Compounding this epigenetic dysregulation is a redox-driven metabolic shift within the S-adenosyl-L-methionine (SAM) cycle. To meet the demands of antioxidant defense, oxidative stress redirects homocysteine flux away from the remethylation pathway—essential for SAM regeneration—and into the transsulfuration pathway for glutathione biosynthesis (Vitvitsky et al., 2003; Mosharov et al., 2000). This metabolic diversion depletes the cellular methyl donor pool, inducing widespread genomic hypomethylation. Such epigenetic erosion is sufficient to drive chromosomal instability, characterized by loss of heterozygosity and segregation errors, ultimately fueling the genomic evolution of aggressive malignancies (Eden et al., 2003).

Global hypomethylation, however, coexists with site-specific hypermethylation at key regulatory loci. Although SAM depletion disrupts epigenetic integrity (Mentch et al., 2015), a targeted recruitment mechanism allows specific tumor suppressors to sequester the limited remaining methyl pool. A convergence of toxic drives this: oxidative damage recruits DNMT-containing silencing complexes to damaged chromatin (O'Hagan et al., 2011), while activated AhR signaling directs these complexes particularly to the promoters of *BRCA1* (Papoutsis et al., 2010; Papoutsis et al., 2012).

This synergistic recruitment enforces dense silencing of cytoprotective genes, overriding the global hypomethylation trend (Romagnolo et al., 2015). Crucially, this targeted suppression extends to the cellular antioxidant defense itself, with the mitochondrial antioxidant superoxide dismutase 2 (SOD2) being a principal target (Cyr et al., 2013). Oxidative damage directs these silencing complexes to GC-rich promoter regions (O'Hagan et al., 2011), a mechanism likely underlying the specific SOD2 hypermethylation observed in breast tumorigenesis (Hitchler et al., 2014). The resulting depletion of SOD2 amplifies the initial oxidative stress, creating a self-perpetuating cycle that sustains the repressive epigenetic state (Cyr et al., 2013).

Ultimately, this metabolic–epigenetic axis dictates subtype-specific tumor evolution. Although luminal phenotypes maintain reliance on estrogenic signaling, ER-negative and triple-negative subtypes develop a critical dependence on constitutive AhR signaling (Romagnolo et al., 2015). Mechanistically, AhR activation transcends metabolic adaptation; it directly recruits epigenetic silencers to differentiation-related promoters, enforcing a site-specific blockade that arrests cellular maturation (Romagnolo et al., 2015). This sustained repression establishes a permissive environment for the acquisition of stem-like traits. Consistent with the established role of the AhR pathway in regulating cancer stem cell plasticity (Akhtar et al., 2022), this signaling axis provides the molecular scaffold supporting the dedifferentiated, aggressive phenotype characteristic of triple-negative breast cancer.

### 4.3 Evolution of malignant phenotypes: from stemness to resistance

The convergence of metabolic reprogramming and oxidative–epigenetic dysregulation drives a shift in tumor plasticity, transitioning the cellular response from molecular injury to active malignant progression. This evolutionary trajectory varies across breast cancer subtypes, dictated by the interplay between specific PCB toxicological profiles and intrinsic cellular vulnerability thresholds.

In TNBC, AhR agonists function as active drivers of cellular plasticity. Unlike ER-positive phenotypes, TNBC lacks the dependency on estrogen signaling, rendering it uniquely susceptible to environmental AhR ligands, such as dioxin-like compounds, that exploit the frequently overexpressed AhR (Romagnolo et al., 2015; Stanford et al., 2016). Here, the AhR acts beyond its canonical role as a xenobiotic sensor; it functionally converges with the Wnt/β-catenin pathway and directly regulates pluripotency factors such as ALDH and Sox2 (Stanford et al., 2016; Al-Dhfyan et al., 2017). This signaling axis enforces a dedifferentiated, stem-like phenotype, which is mechanistically linked to the aggressive metastasis and high recurrence rates characteristic of this subtype (Chatterjee and Banerjee, 2023; Goode et al., 2013). Thus, for TNBC, exposure to dioxin-like congeners may not merely promote growth but potentially select for a stem-cell-enriched population capable of surviving conventional chemotherapy.

Conversely, estrogen receptor-positive (ER+) tumors evolve under a “conflict-driven” selective pressure. These cells face a hostile microenvironment created by the simultaneous presence of estrogenic (Group I) and enzyme-inducing (Group III) congeners (Wolff et al., 1997). Although Group I congeners provide mitogenic stimuli (Safe, 1994), the metabolic hyperactivity induced by Group III congeners generates a persistent flux of reactive quinones and ROS (Grimm et al., 2015). We propose that this oxidative toxicity creates a specific evolutionary bottleneck. The physiological conflict between the estrogenic drive observed in Group I and the oxidative stress induced in Group III functionally mimics a state of chronic endocrine disruption. This pressure penalizes cells that are strictly dependent on canonical estrogen signaling, favoring the acquisition of intrinsic ligand independence. Although *ESR1* mutations are canonically associated with post-treatment resistance (Khan et al., 2025; Crucitta et al., 2023), environmental stress drives a functionally convergent phenotype. By mandating adaptation to an unstable hormonal and redox milieu, this exposure selects for subclones with reduced estrogen dependency or for those that activate alternative pathways. Consequently, the tumor is biologically “primed” to evade therapeutic blockade before clinical intervention.

These divergent evolutionary histories dictate distinct therapeutic vulnerabilities. For TNBC, the reliance on AhR-mediated stemness represents a druggable target; pharmacological disruption of the AhR axis has been shown to dismantle resistance programs (Baker et al., 2019). Conversely, the biology of ER+ lineages is characterized by a high intrinsic oxidative burden. This is evidenced by significantly elevated accumulation of oxidative DNA lesions (e.g., 8-OHdG) in ER+ tumors compared to ER− subtypes (Musarra et al., 1996), implying a potentially precarious reliance on antioxidant defense mechanisms in resistant subclones. This “ROS addiction” renders them paradoxically hypersensitive to pro-oxidant therapies. Agents such as piperlongumine or high-dose vitamin C can exploit this liability by elevating intracellular ROS levels beyond the lethal threshold, thereby inducing selective apoptosis (Uetaki et al., 2015; Raj et al., 2011).

## 5 Comprehensive public health interventions for polychlorinated biphenyls

**FIGURE 3:**
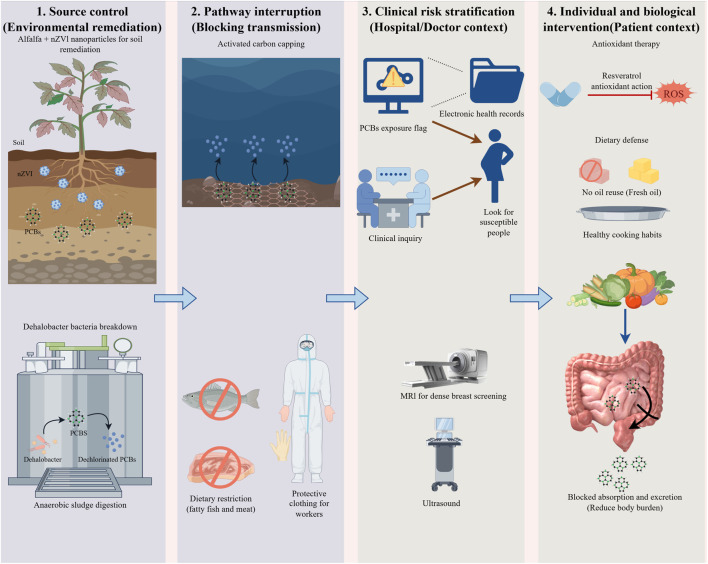
Multilevel intervention framework for PCB-associated breast cancer risk management.

Preceding sections have established that specific PCB congeners drive breast cancer pathogenesis through defined endocrine and epigenetic mechanisms. However, translating this biological insight into effective prevention remains hindered by systemic barriers. Despite global restrictions, the convergence of legacy environmental persistence and inadvertent contemporary production sustains human exposure at biologically active levels. Fragmented regulatory measures fail to address this reality; effective mitigation mandates a multi-scalar strategy spanning the entire pollutant lifecycle. We, therefore, propose an integrated intervention framework (Figure 3). By coordinating upstream source control with downstream protection of susceptible populations, this approach aims to systematically reduce cumulative toxic burdens and attenuate the risk of PCB-associated malignancy.

Figure 3 illustrates an integrated strategy linking environmental remediation to clinical precision. Upstream, source-control measures combine bio-nano remediation (nZVI/alfalfa) and microbial dechlorination to reduce pollutant bioavailability. Downstream, clinical protocols leverage exposure history to stratify patients. At the same time, individual interventions—particularly dietary defense and targeted antioxidant therapy (e.g., resveratrol)—are implemented to block absorption, facilitate excretion, and neutralize oxidative stress. This synthesis establishes a continuous “source-to-patient” defense system, advancing prevention from static hazard assessment to active risk mitigation.

### 5.1 Source control

Effective risk management demands the containment of persistent environmental reservoirs, particularly informal electronic waste processing zones—primary drivers of continued PCB emissions (Montano et al., 2022; Robinson, 2009). As the International Agency for Research on Cancer classifies PCBs as Group 1 carcinogens, reducing environmental loads is critical to maintaining human exposure within tolerable limits (Lauby-Secretan et al., 2013; Chain et al., 2018). Remediation strategies are shifting toward bio-nano synergistic systems to address complex contamination profiles. Wu et al. (2021) characterized this mechanism using an nZVI-alfalfa “rhizosphere reactor.” The nanomaterials reprogram root metabolism, stimulating the exudation of specific amino acids. These metabolites selectively enrich PCB-oxidizing bacteria, notably *Dyella*, driving accelerated degradation through enhanced plant–microbe interactions. Wang et al. (2023) expanded this biological framework to co-contaminated matrices. Their work demonstrates that a clover–*Rhizobiales* association drives the simultaneous remediation of PCBs and cadmium, validating the versatility of plant–microbe synergism in complex soil environments. Beyond recruiting indigenous taxa, recent studies have advanced by exploring the metabolic potential of recovered microbes. Lin et al. (2024) demonstrated that co-cultivating specific resuscitated strains (e.g., *Pseudomonas* sp. and *Achromobacter* sp.) significantly enhances PCB biodegradation efficiency and intermediate mineralization compared to individual strains, attributing this to complementary metabolic pathways.

However, a critical limitation remains for highly chlorinated congeners, which are inherently recalcitrant to direct aerobic attack. As highlighted in a recent review by Hashmi and Mughal (2025), anaerobic reductive dechlorination is the initial step in removing chlorine atoms from the biphenyl ring. However, this process is kinetically slow, limiting overall remediation efficiency. To address this issue, Han et al. (2024) used anaerobically digested sludge to enrich organohalide-respiring bacteria. This biostimulation strategy significantly enhanced the reductive dechlorination of highly chlorinated PCBs by promoting taxa such as *Dehalobacter* and *Dehalobium*. Complementing such biological optimization strategies with chemical methods to ensure complete detoxification, Wu et al. (2022) developed the Bio-RD–PAOP system. This integrated approach couples microbial reductive dehalogenation with persulfate-based advanced oxidation, effectively converting recalcitrant congeners into lower-chlorinated intermediates that are susceptible to radical attack, thereby completing the loop of extensive mineralization.

### 5.2 Pathway interruption

Interventions must intercept PCB migration at critical interfaces to prevent mobilization from environmental reservoirs into biological systems. In organic-rich aquatic sediments, sediment gas ebullition can drive contaminant resuspension even through physical barriers (Dahlberg et al., 2023). Although physical stabilization, such as capping, mitigates particle movement (Dahlberg et al., 2023), its limitations in retaining dissolved congeners underscore the need for chemical sequestration as a complementary approach. Wang et al. (2022) demonstrated that *in situ* activated carbon amendment reduces PCB bioavailability by 80%–90% over 7 years, effectively trapping contaminants within the sediment matrix. Recent advancements favor active removal over passive sequestration. Muller et al. (2025) developed tailorable superparamagnetic iron oxide nanoparticles functionalized with phosphonic acid derivatives. This magnetic extraction strategy demonstrates high efficiency in aqueous matrices, achieving up to 97.6% removal of PCB 14 and incorporating specific electrostatic mechanisms to target the highly toxic congener PCB 77 (Muller et al., 2025).

For terrestrial–aquatic interfaces, bio-enhanced interception offers a dynamic barrier. Ma et al. (2023) established a methodology utilizing biochar-immobilized microorganisms within bio-permeable reactive barriers. This system couples physical adsorption with microbial degradation, effectively intercepting soluble pollutants in contaminated soils or groundwater before they diffuse into broader ecosystems. At the air–water interface, bio-augmentation curbs volatilization. Ramotowski et al. (2025) reduced vapor-phase accumulation of lower-chlorinated congeners by 54%–60% using the aerobic degrader *Paraburkholderia xenovorans* LB400, directly mitigating inhalation risks for adjacent communities.

Beyond environmental remediation, reducing human body burden requires actively interrupting trophic transmission through strict behavioral and surveillance measures. Historically, the dominant exposure route, dietary intake, requires management strategies such as those implemented in the Brescia cohort, where serum PCB levels decreased by ∼50% over a decade following restrictions on local agricultural consumption (Magoni et al., 2016). Effective surveillance, however, must transcend concentration metrics. The WHO Toxic Equivalency Factor (TEF) framework weights congeners by potency relative to TCDD, prioritizing the elimination of biologically active fractions rather than the simple reduction of mass (Van den Berg et al., 2006). Finally, blocking para-occupational exposure necessitates rigorous on-site decontamination to prevent contaminant transport from industrial zones to households via clothing (Kaifie et al., 2019). Synthesizing these findings, integrating environmental containment with vector disruption establishes a robust, multi-layered defense system.

### 5.3 Protection of susceptible populations

Susceptibility to PCB-induced carcinogenesis varies across populations. Early epidemiological studies focused on genetic polymorphisms, such as *CYP1A1* variants, to account for heterogeneity in risk (Li et al., 2005). Contemporary research, however, identifies epigenetic modifications as a critical mediator between exposure and effect. In the Anniston Community Health Survey, Pittman et al. (2020) associated cumulative PCB exposure with differentially methylated regions enriched in immune-related genes, suggesting that PCBs perturb immune epigenetic profiles. The tumor microenvironment (TME) also plays a pivotal role. Recent reviews highlight that persistent pollutants, including PCBs and dioxins, may modulate the TME—such as by altering endothelial integrity or stromal interactions—to drive EMT, potentially facilitating metastasis (Koual et al., 2020).

However, these cellular vulnerabilities do not exist in isolation; the external exposure landscape substantially compounds them. Structural determinants exacerbate these biological risks by dictating the cumulative toxic burden. James-Todd et al. (2016) highlighted that racial and ethnic minorities face a disproportionate burden of endocrine-disrupting chemicals due to systemic socioeconomic and health disparities. Structural determinants exacerbate these biological risks. A comprehensive analysis by Nguyen et al. (2020) of U.S. women demonstrated that racial and ethnic disparities in chemical body burdens persist even after rigorous adjustment for socioeconomic status (SES). These systemic inequities intersect with lifestyle and dietary patterns in nuanced ways. Although traditional models often link lower SES to heightened environmental risks, Liu Y. et al. (2023) utilized advanced statistical modeling of NHANES data to reveal a positive correlation between PCB concentrations and both age and income. This suggests a “socioeconomic paradox” for certain persistent pollutants: higher SES may facilitate greater access to contaminated, lipid-rich foods, such as high-trophic level seafood, thereby increasing the body burden through specific dietary choices (Liu Y. et al., 2023). This intersection of demographics and economic lifestyle underscores the complexity of exposure modeling beyond simple SES metrics.

Structural inequities represent only one dimension of risk; the developmental timing of exposure is equally definitive. Recent high-quality evidence, including a comprehensive systematic review by Balalian et al. (2024) and cohort findings by Fábelová et al. (2025), definitively links prenatal PCB exposure to cognitive deficits and reduced IQ scores, thereby challenging historical assumptions about safe exposure levels during development. Consequently, current safety assessments may underestimate risks during critical developmental windows. Addressing these uncertainties, the American College of Obstetricians and Gynecologists invokes the precautionary principle, advocating minimized exposure to environmental agents during pregnancy even in the absence of definitive toxicity data (American College of Obstetricians and Gynecologists’ Committee on Obstetric Practice, 2021). Policy frameworks must, therefore, evolve to prioritize these sensitive physiological stages over general population averages.

## 6 PCBs and the clinical management of breast cancer

### 6.1 Risk identification and stratification

Translating environmental awareness into clinical action requires systematic screening protocols. Accordingly, standardized frameworks, such as the “I-PREPARE” model, are used to systematically inventory exposure history (Paranzino et al., 2005). Integrating these frameworks into electronic health records has the potential to optimize workflows by flagging high-risk residence history and dietary patterns during critical susceptibility windows (Wood et al., 2025; Akras et al., 2025). For patients with considerable exposure histories, quantitative serum biomonitoring is the definitive step to distinguish background environmental exposure from elevated body burdens by comparing results against established reference values (Iavicoli et al., 2019). However, quantifying body burden alone provides limited insights into individual biological consequences. To address this, risk stratification is evolving from static genetic profiling to dynamic molecular surveillance. Although historical epidemiologic frameworks primarily focused on single-gene polymorphisms, such as *CYP1A1* (Li et al., 2005), recent integrative network toxicology analyses have highlighted specific signaling hubs—notably EZH2 and AKT1—as pivotal mediators of PCB-induced carcinogenic potential (Yang et al., 2025). The dysregulation of these targets correlates with distinct immune infiltration patterns and tumor aggressiveness *in silico*, positioning them as candidate “effect biomarkers” for monitoring the biological progression from environmental exposure to neoplastic perturbation (Yang et al., 2025).

This biological progression is paralleled by macroscopic tissue changes; epidemiological evidence links elevated serum PCB burdens to increased mammographic density, particularly among younger women (Rusiecki et al., 2020). Because dense tissue creates a “masking effect” that significantly compromises mammographic sensitivity—decreasing below 50% in extremely dense breasts—supplemental imaging is often clinically indicated (von Euler-Chelpin et al., 2019). However, access to these diagnostic resources is markedly stratified. Recent large-scale cohort data reveal that utilization rates for supplemental ultrasound and MRI remain negligible (2.8% and 0.3%, respectively) and are concentrated among women with private insurance and higher socioeconomic status (Foster et al., 2025). Furthermore, despite the proliferation of density notification laws, structural barriers—including geographic isolation and limited health literacy—continue to impede effective follow-up for underserved populations (Fazeli et al., 2025; Kressin et al., 2021). Thus, screening protocols must move beyond biological risk assessment to address the systemic disparities that dictate diagnostic equity.

### 6.2 Navigating dietary risks in a global context

Dietary intake, particularly of aquatic products, remains a primary route of PCB exposure, necessitating a balance between nutritional benefits and contaminant risks (Donat-Vargas et al., 2020). However, effective risk management requires a holistic view of the food web as bioaccumulation is not exclusive to seafood (Rahul et al., 2024). A 2024 systematic analysis indicates that although marine crustaceans (e.g., mud crabs) exhibit the highest contaminant peaks, terrestrial staples such as dairy and meat are critical vectors due to their dominance in global diets and susceptibility to feed-borne contamination (Rahul et al., 2024). Consequently, a broad avoidance of fish is clinically counterproductive; it forfeits established cardiovascular protection without effectively eliminating the total dietary PCB burden derived from terrestrial sources (Donat-Vargas et al., 2020; Rahul et al., 2024).

The final pollutant burden is not solely determined by food source; culinary processing plays an equally critical role in modulating exposure levels. Preparation methods significantly alter final pollutant burdens. Thermal processing, such as frying, can theoretically reduce PCB levels through fat loss and volatilization; however, commercial practices involving repeated oil use may promote PCB accumulation, resulting in higher concentrations than those observed with grilling or fresh-oil preparation. Domestic cooking practices that prioritize lipid separation and avoid oil reuse serve as a primary defense against dietary exposure (Abhishek and Agarwal, 2025).

Beyond physical reduction, dietary bioactive compounds offer a mechanism for chemical defense. Resveratrol mitigates oxidative stress and cytotoxicity induced by ortho-substituted congeners, such as PCB 153. However, this protection is structure-dependent, showing limited efficacy against dioxin-like congeners such as PCB 77, suggesting that chemoprevention requires congener-specific tailoring (Miletic et al., 2023).

### 6.3 Post-diagnosis prognosis and intervention

In the post-diagnostic setting, PCB load may function as an adverse prognostic factor, particularly in the short term. Prospective cohort data associate higher baseline concentrations of congeners PCBs 74, 99, and 118, with significantly increased 5-year breast cancer-specific mortality (Parada et al., 2020), suggesting that highly exposed patients may benefit from intensified surveillance during the early survivorship period. Furthermore, epidemiological evidence suggests a potential interplay between pollutant exposure and metabolic factors. Chronister et al. (2021) reported that the risk of all-cause mortality associated with elevated serum PCBs is exacerbated by high dietary acid load. Patients with high PCB levels consuming acid-forming diets (e.g., high meat/dairy and low plant intake) exhibited the poorest overall survival.

For cases where lifestyle modification is insufficient to manage exceptionally high body burdens, active therapeutic reduction remains an experimental but feasible frontier. Controlled studies demonstrate that the non-absorbable fat olestra accelerates PCB excretion, supporting a “block absorption-promote excretion” strategy (Jandacek et al., 2014). Although not yet standard of care, such physiological elimination protocols warrant evaluation for patients with exceptionally high body burdens under strict supervision. Transitioning from passive observation to active management represents a necessary evolution in environmental medicine. Future clinical protocols must standardize interventional thresholds, ensuring that toxicokinetic data, genetic susceptibility, and individual prognostic risk inform therapeutic decisions.

## 7 Conclusions and perspectives

Specific PCB congeners function as distinct drivers of breast carcinogenesis. This pathogenicity stems from the convergence of endocrine disruption and AhR activation, which trigger metabolic reprogramming and oxidative stress, thereby enforcing the epigenetic silencing of tumor-suppressor genes. Critical developmental windows, genetic background, and cumulative environmental burdens further modulate individual susceptibility. However, methodological heterogeneity across epidemiological studies currently constrains risk interpretation. Inconsistent congener panels and disparate lipid-adjustment strategies compromise the precision of meta-analyses. Furthermore, the epidemiological reliance on parent compounds likely underestimates risks driven by reactive metabolites identified in experimental models. Future inquiry must, therefore, shift from qualitative hazard identification to quantitative risk assessment. Capturing exposure dynamics requires longitudinal cohorts spanning the whole life course—from prenatal development to menopause—alongside standardized profiling of both parent compounds and metabolites. To resolve synergistic effects often masked by single-pollutant models, advanced mixture analyses, such as quantile-based frameworks, are essential. Mechanistically, research must prioritize subtype-specific vulnerabilities, particularly the link between AhR-active congeners and triple-negative phenotypes. Finally, effective risk mitigation must originate upstream. Reducing the global pollutant load requires integrating environmental engineering—particularly bio-nano remediation and pathway interception—with public health policies that address structural inequities. Protecting susceptible populations during critical developmental windows establishes the baseline for clinical success. This environmental control ultimately empowers clinical management, enabling risk stratification and intervention within a comprehensive “source-to-patient” precision prevention framework.
