# Correction: Polychlorinated biphenyls and breast cancer evidence, mechanisms, and risk management

**DOI:** 10.3389/fcell.2026.1826345

**Published:** 2026-04-09

**Authors:** Shuaiyang Zhang, Hongliang Cao, Runzhou Lian, Ying Liu, Yuting Zhu, Xinxin Gui, Tianzi Kong, Aiping Shi

**Affiliations:** 1 Department of Breast Surgery, General Surgery Center, The First Hospital of Jilin University, Changchun, China; 2 Department of Urology, The First Hospital of Jilin University, Changchun, China

**Keywords:** breast cancer, epigenetic locking, metabolic reprogramming, polychlorinated biphenyls, precision prevention

An incorrect number was provided for the Science and Technology Development Plan Project of the Science and Technology Department of Jilin Province. The correct number is 20250204057YY.

The original article has been updated.

